# Ginsenoside Rb3 Mitigates Murine Ulcerative Colitis by Modulating Intestinal Microflora and Short-Chain Fatty Acids

**DOI:** 10.4014/jmb.2508.08035

**Published:** 2026-01-22

**Authors:** Wei Zhang, Qiben Wang, Tianjie Zhang, Yanbing Meng, Chuyu Lin, Siyu Zeng, Qiujuan Ou

**Affiliations:** 1 Department of Physiology, School of Basic Medicine, Xiangnan University, Chenzhou, Hunan 423000, P.R. China; 2 Journal Editorial Department, Xiangnan University, Chenzhou, Hunan 423000, P.R. China; 3 Department of Physiology, School of Basic Medicine, Xiangnan University, Chenzhou, Hunan 423000, P.R. China; 4 Department of Human Anatomy, School of Basic Medicine, Xiangnan University, Chenzhou, Hunan 423000, P.R. China; 5 Clinical Medicine from Junior College to Bachelor, Xiangnan University, Chenzhou, Hunan 423000, P.R. China; 6 Department of Nephrology and Rheumatism Immune Disease, Affiliated Hospital of Xiangnan University, Xiangnan University, Chenzhou, Hunan 423000, P.R. China

**Keywords:** Ginsenoside Rb3, Ulcerative colitis, Intestinal bacteria, Short-chain fatty acids

## Abstract

Ecological dysregulation leads to the progression of inflammatory bowel disease (IBD). The present study was designed to evaluate whether ginsenoside Rb3 (GR3) ameliorates dextran sulfate sodium (DSS)-induced colitis by modifying the microbiota. The results revealed that GR3 treatment with oral doses of 5 mg/kg suppressed DSS-induced colitis in mice, and its effects were evaluated using a combination of histological analysis, enzyme-linked immunosorbent assays (ELISA), and Western blotting. This was evidenced by a significant attenuation of symptoms such as weight loss, diarrhea, hematochezia, and colonic shortening in the DSS-induced colitis mice. Furthermore, GR3 treatment remarkably elevated the expression of tight junction proteins (occludin and zonula occludens-1) while reducing both inflammatory cell infiltration and inflammatory cytokine concentrations (TNF-α, IL-1β, IL-15, IL-17A, and IL-6). Intriguingly, GR3 treatment also mitigated DSS-induced intestinal dysbiosis by prominently increasing the proliferation of *Lactobacillus* and decreasing the relative abundance of *Bacillus*. Additionally, GR3 treatment significantly modified the metabolism of short-chain fatty acids in colitis mice, especially elevating the levels of acetic acid and butyric acid. These findings suggest that GR3 ameliorates colitis by reshaping the gut microbiota and improving the intestinal barrier and inflammation.

## Introduction

Ulcerative colitis (UC) is a chronic and recurring inflammatory bowel disease characterized by symptoms such as bloody diarrhea and abdominal pain [1]. Diagnosis of UC often involves colonoscopy and histopathological examination, with bleeding diarrhea being a hallmark symptom [2]. The exact etiology of UC remains uncertain, but it is believed to result from a combination of factors, including genetic susceptibility, environmental influences, alterations in enteric immunity, dysbiosis of gut bacteria, abnormal tryptophan (TRP) metabolism, and deficiencies in the epithelial barrier [3]. Notably, research suggests that dysbiosis-induced disruptions in the gut's inflammatory and metabolic processes play a significant role in UC pathogenesis. This is primarily due to decreased microbial diversity, reduced levels of beneficial bacteria, and an increased presence of pathogenic bacteria, leading to an inflammatory response in intestinal genes [4]. Clinical management of UC primarily involves anti-inflammatory and immunomodulatory therapies, often supplemented with biological treatments [5]. However, the current pharmacological treatments for UC frequently carry adverse side effects. In contrast, naturally derived medicines have shown promise in reducing these side effects while effectively treating colitis [6]. Therefore, it is of utmost importance to identify effective natural remedies to minimize side effects and optimize therapeutic outcomes.

The gut microbiota consists of a complex community of bacteria inhabiting the host's intestinal tract, typically categorized into three types: commensal microflora, conditional pathogenic microflora, and pathogenic microflora [7]. Under normal circumstances, a balanced microbial environment is maintained, while any disruption can lead to the overgrowth of pathogenic flora, triggering various enteric disorders [8]. Accordingly, homeostasis of the organism populations serves as an effective natural barrier against pathogenic bacteria. Previous studies have established that alterations of intestinal microflora in UC patients coincide closely with the severity of inflammation [9]. Dysbiosis, the main characteristic of this imbalance, often involves an accumulation of pathogenic bacteria. These bacteria release enterotoxins that increase the permeability of the intestinal epithelium, allowing luminal bacteria and their by-products to penetrate the lamina propria of the intestinal mucosa. This contributes to mucosal immune dysregulation and compromises the intestinal barrier, ultimately shifting the diversity and composition of the gut microbiome [10]. Short-chain fatty acids (SCFAs), which are metabolites produced by the gut microbiota, play a crucial role in UC therapy. SCFAs serve various functions, including providing energy, protecting the intestinal barrier, and modulating intestinal inflammation [11]. In UC therapy, SCFAs have been recognized as a signaling element to activate G protein-coupled receptors (GPCRs) on the cell surface and suppress histone deacetylases through substrate transport proteins within the cell to accomplish anti-inflammatory purposes [12]. Intriguingly, numerous preclinical investigations have documented the beneficial impact of targeted modulation of intestinal microflora in the management of ulcerative colitis, albeit the underlying mechanisms are still to be unraveled [13-16]. Therefore, by reshaping the intestinal microecology and restoring the intestinal mucosal barrier, a novel potential therapeutic avenue may be proposed for the treatment of UC patients.

**Fig. 1:**
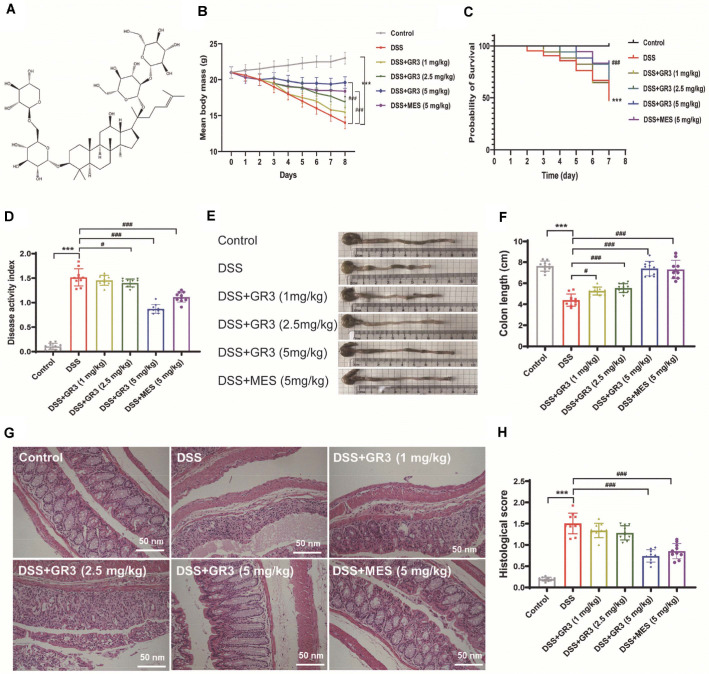
Ginsenoside Rb3 ameliorated the symptoms of DSS-induced chronic colitis in mice. Animals were randomly divided into six groups and the ulcerative colitis mouse model in this experiment was initiated with DSS. (**A**) Chemical structure of Ginsenoside Rb3. (**B**) Changes in body weight. (**C**) The probability of survival of mice in mice. (**D**) The DAI scores of mice. (**E-F**) Macroscopic view and quantitation of colon length. (**G**) The mouse intestinal tissue of H&E staining (200 ×). (**H**) Quantitation of histological scores of mice. All data are expressed as the mean ± SD (n = 10). *** *P* < 0.001. ^#^ *P* < 0.05, ^##^ *P* < 0.01, ^###^ *P* < 0.001.

Ginseng (scientific name: Panax ginseng C. A. Meyer), a perennial herb of the family Cinchocaine, is a genus of ginseng. As a strengthening tonic, the fleshy roots of ginseng apply in regulating blood pressure, restoring heart function, and alleviating neurasthenia and physical weakness, whilst expectorating, stomachic, diuretic, and euphoric [17]. Ginsenosides, a major active ingredient of ginseng, primarily exists in the roots, flower buds stems and leaves of ginseng, as well as in the roots, stems, and leaves of American ginseng or Panax ginseng, of which ginsenoside Rb3 (GR3) serves as a major pharmacological active element of ginseng, American ginseng and Panax ginseng [18]. In this study, “ginseng” refers to the genus Panax; more specifically, the term “Panax ginseng” denotes the Asian species Panax ginseng, whereas “American ginseng” denotes the North American species *P. quinquefolius*. Although both species share the genus Panax and contain dammarane-type ginsenosides, they exhibit distinct quantitative ginsenoside profiles. For example, Panax ginseng is generally characterized by a relatively higher Rg1/Rb1 ratio, whereas *P. quinquefolius* shows a profile enriched in Rb1-type ginsenosides [19, 20]. As the fundamental parent nucleus of GR3 is a dammarane-type tetracyclic triterpene, which belongs to the proto-ginseng diol-type triterpene saponin. Contemporary research has documented that GR3 exerts extensive biological activities encompassing anti-tumor, anti-inflammatory, antioxidant, and anti-apoptotic pharmacological effects [21]. As saponins, oral absorption through the intestinal tract is comparatively inferior while most of them require metabolism by enteric bacteria before their metabolites enter the body [22]. At this point, the pharmacological effects of ginsenosides are inextricably intertwined with the metabolism of intestinal bacteria. Intriguingly, the studies of Huang *et al*. [23] have revealed that GR3 (illustrated in Fig. 1A) diminished polyps formation accompanied by reshaping the intestinal microflora and intestinal microenvironment in ApcMin/+ mice, hinting that GR3 probably exerts a modulatory role in ulcerative colitis and intestinal microflora.

To address how a single ginsenoside can selectively reshape the microbiome, this study evaluates GR3 within a host–microbe framework, integrating barrier integrity, inflammatory tone, and fermentative metabolism. We also position GR3 among other ginsenosides reported in experimental colitis to delineate class-shared versus GR3-specific features. At present, prominent mitigating effects of natural polysaccharides on ulcerative colitis have been investigated in the literature [24]. For instance, some papers have indicated that the other monomers of ginsenoside alleviated the symptoms of colitis in mice [25-28] whereas the mechanisms underlying the beneficial effects of GR3 are poorly understood [29]. Our previous work has discussed the beneficial properties of GR3 on enteropathy. Hence, by gavage of GR3 in mice with colitis, we have explored possible mechanisms involved in the therapeutic effects of GR3 on colitis, aiming to facilitate the preparation of functional foods or nutraceuticals.

## Materials and Methods

### Chemicals and Reagents

Ginsenoside Rb3 (Product code: B21052, purity: 97%) was supplied from Shanghai Yuanye Bio-Technology Co., Ltd., (China) and the chemical structure is illustrated in Fig. 1A. The creatinine (CR) kit (C011-2-1), blood urea nitrogen (BUN) kit (C013-2-1), alanine aminotransferase (ALT) kit (C009-2-1) and aspartate aminotransferase (AST) kit (C010-2-1) were acquired from Nanjing Jiancheng Institute of Biological Engineering. Primary antibodies of Occludin (ab216327), Zona occludens 1 (ZO-1) (ab221547) and GAPDH (ab8245) were purchased from Abcam (UK). Primer was synthesized by Shenzhen Huada Gene Co., while fecal occult blood test kit (JT9068) was purchased from Beijing Emo Biotechnology Co.(China).

### Animals

A total of 120 male C57BL/6 mice (6–8 weeks, 18–22 g) were purchased from Shanghai Siple-Bikai Laboratory Animal Co. (China). The mice were housed under controlled conditions with a temperature of 22 ± 2°C, a relative humidity of 50 ± 10%, and a 12-h light/dark cycle. An adequate supply of food and water was provided to guarantee that all rodents were freely fed and watered to their satisfaction. All animal experimental procedures were reviewed and approved by the Animal Ethics Committee of Xiangnan University (protocol number DW2021–02–01) and were conducted in accordance with the National Institutes of Health Guide for the Care and Use of Laboratory Animals.

Mice were euthanized using CO_2_ inhalation following a standardized protocol. The animals were placed in a CO_2_ euthanasia chamber, and the CO_2_ valve was gradually opened to allow a slow and controlled increase in carbon dioxide concentration. Once the mice lost consciousness, the CO_2_ concentration was increased to 100%. Unconsciousness was confirmed by the absence of reflexes, such as the lack of a toe pinch response and loss of muscle tone. CO_2_ flow was continued for an additional 2 min to ensure that the mice were fully deceased with no vital signs remaining.

### DSS-Induced Ulcerative Colitis Model

As pathological alignments of the DSS-induced ulcerative colitis model in murine were similar to those of human patients with ulcerative colitis, the ulcerative colitis mouse model in this experiment was initiated with DSS. Upon acclimatized feeding for one week, the mice were randomly divided into 6 groups (20 mice per group) depending on body weight, respectively, blank group (Control), model group (DSS), mesalazine (MES, 5 mg/kg) positive group (DSS + Mes 5 mg/kg), low dose GR3 administration DSS+GR3 (1 mg/kg), middle dose GR3 administration DSS+GR3 (2.5 mg/kg), and high dose group of ginsenoside administration Rb3 DSS+ GR3 (5 mg/kg). As previously reported [30], the ulcerative colitis murine model was evoked with a 4% DSS (MP Biomedical) drinking water for 7 consecutive days, followed by distilled water for 1 day. GR3 was administered to the mice via oral gavage. The treatment included three different dosages: 1 mg/kg, 2.5 mg/kg, and 5 mg/kg, with a single oral gavage given daily throughout the treatment period. The positive control drug (MES) was also administered by oral gavage at a dose of 5 mg/kg, with daily gavage during the treatment period. Both GR3 and MES treatments commenced on day 1 of the experiment and continued through day 7, with daily administration for the entire duration. Symptoms including weight, food intake, mental activity, diarrhea, and blood in stool were registered daily to investigate the anti-ulcerative colitis activities of GR3. Particular attention was given to humane endpoints such as significant weight loss (>20%), persistent rectal bleeding, and severe lethargy. Animals exhibiting any of these severe symptoms were promptly euthanized via CO_2_ inhalation to prevent unnecessary suffering.

### Assessment of Intestinal Permeability

To summarize, fluorescein isothiocyanate (FITC)-dextrose (4 kDa; Sigma-Aldrich, USA) at a concentration of 100 mg/ml was solubilized in PBS at a concentration of 100 mg/ml was solubilized in PBS. Following fasting on day 7, animals were gavaged orally with FITC -dextrose after a 4 h fast. A blood sample was collected after 4 h and centrifuged at 1,000 rpm/min for 20 min. Subsequently, supernatants were recovered for fluorescence quantification at excitation wavelengths of 485 nm and 535 nm with Multiskan FC Microplate Photometer (Thermo Fisher Scientific, USA).

For observing the therapeutic effect of GR3 on the improvement of colitis, intestinal permeability was denoted by detecting the level of Lipopolysaccharides (LPS) in the serum. For simplicity, after leaving the blood to clot naturally for 15 min and centrifuging for about 20 min (2,000-3,000 rpm/min), the supernatant is collected. According to the instructions of the kit, chromogenic solution, and termination solution were added, white wells were zeroed, and the absorbance (OD) of the samples was measured at 450 nm with Multiskan FC Microplate Photometer (Thermo Fisher Scientific).

### Assessment of Disease Activity Index (DAI)

Scoring of body weight, fecal viscosity, and bleeding in the feces of animals is performed after DSS administration, as described by Veenstra *et al*. [31]. Disease Activity Index (DAI) scores were documented daily. According to the instructions, the blood in feces were determined with a hemoccult test (hemo FEC, Roche, Germany). Scoring of body weight, feces viscosity, and bleeding in the feces of animals is performed after molding. DAI scores were documented before mouse life was devoted with CO_2_ to evaluating the severity of colitis. A rating of 0 to 4 for each index was assigned, 0 for no signs of disease and 4 for significant sickness, as listed in Table 1.

### H&E Staining and Pathological Assessment

Following the experiment, all mice were euthanized before colon tissue was collected. Intestinal morphology was visualized by visual inspection as well as measured for the length of the colon. Subsequently, after removing the contents and washing the intestinal lumen with saline, an intersection of 2 cm of the rectum near the colon was fixed in 4% formaldehyde. The remaining sections were refrigerated at -80oC and preserved for subsequent experiments. Fixation was terminated by flushing with running water with 4% formaldehyde solution, the colonic tissues were dehydrated with varying concentrations of ethanol, conditioned in xylene solution, wrapped in paraffin, sliced, and stained with H&E. Ultimately, the pathological changes of intestinal lesions were then monitored and scored by professional pathologists.

Scores for pathological injury were as follows: 0 for the rarity of infiltrating cells in the lamina propria; 1 for an observed disturbance of the granular cells in the lamina propria; 2 for rendezvous of inflammatory cells into the submucosa; in addition, 3 for an expansion of the inflammatory infiltrate over the entire wall. In contrast, hermitage impairment was scored from 0, with the integrity of the hermitage; 1, with the absence of 1/3 of the base; 2, with the absence of 2/3 of the base; or 3, with the destruction of the entire basement.

### Liver Function Analyses

According to preliminary research published in Clinical Gastroenterology and Hepatology, the treatment of inflammatory bowel disease (IBD), such as ulcerative colitis and Crohn's disease, as well as its treatment has a potential for increasing the risk of liver injury and chronic kidney disease [32, 33]. Briefly, after natural clotting of the blood for 15 min, it was centrifuged for 15 min (3,000 rpm/min) and its supernatant was collected. The absorbance of the samples was measured sequentially by adding different solvents in sequence according to the instructions of the kit.

### Real-Time Quantitative PCR

Simply, with the extraction of total RNA from the tissue with the synthesis of first strand cDNA following a kit from Toyobo Corp., Step 1 real-time quantitative PCR was applied with the following procedure: 95oC for 3 min, 40 cycles of 95oC min for 10 sec each followed by 60oC seconds for 30 sec. Gene-expression levels were normalized for beta-actin. Calculation of the relative level of each target gene mRNA transcript to the control utilizing the comparative Ct (ΔCt) method. Primer availability is shown in Table 2 for sequences (5′ to 3′).

### Western Blotting

The procedure for protein blotting experiments was performed as previously described [34]. To simplify, the proteins in the colonic tissue were extracted with lysis buffer with the bicinchoninic acid assay (BCA) kit for quantification. Sodium dodecyl sulfate-polyacrylamide gel electrophoresis (SDS-PAGE) gels were employed to dissociate and transfer the protein samples onto PVDF membranes. Subsequently, blocking with 3% bovine serum albumin (BSA) for 2 h at room temperature before incubation with primary antibodies overnight, followed by the incorporation of secondary antibodies and detection with ChemiDoc MP Imaging System (Bio-Rad, USA).

### Enzyme-Linked Immunosorbent Assay

After centrifugation at 3000 r/min for 10 min at 4oC, serum extracted from the eyes of mice was stored at -80oC for biochemical analysis. Following the procedure of the ELISA kit, levels of inflammatory cytokines (Interleukin 4 (IL-4), Interleukin 1β (IL-1β), Interleukin 15 (IL-15), Interleukin 17A (IL-17A), Interleukin 6 (IL-6), Interleukin 10 (IL-10), Transforming Growth Factor-β1 (TGF-β1) and Tumor necrosis factor-α (TNF-α)) were recorded from serum and enteric tissues of all mice.

### 16S rDNA Sequencing

Inflammatory bowel disease originates from the invasion of enteric symbiotic organisms and their metabolites towards the intestinal barrier, dynamically driving the activation of inflammatory cells. Gut bacteria from mouse feces were profiled by NovoMagic Ltd., based on 16S rDNA sequence alignment, aiming to ascertain the impact of EDA on DSS-induced murine colitis. The primers for the 16SrDNA V3-V4 region were designed to amplify the 16SrDNA fragments by PCR and purified, then the concentration of the purified PCR amplicons was determined by Qubit2.0. V3–V4 16S rRNA gene sequencing has limited resolution for Bacillus sensu lato; accordingly, we report this signal at the genus level with family/order context (*Bacillaceae*/*Bacillales*) and avoid species-level inference. The cDNA libraries were constructed and sequenced by the Illumina-MiSeq platform. The primers for PCR amplification were F:5'-NNNNCCTACGGGGGNGGCWGCAG-3'; and R:5'-NNNNGGACT ACHVGGGGTATCTAATCC-3'. Clustering analyses of the original sequencing data were performed by USEARCH software with 97% similarity of operational taxonomic units (OTUs). α-diversity analysis was applied with Chao1 indices, while β-diversity analysis was conducted with principal coordinates analysis (PCoA) and Non-metric multidimensional scaling (NMDS). The composition of intestinal bacteria was measured at the phylum and genus levels, and the Heatmap presented the species composition and abundance information in a color gradient. The similarity and variability of community composition at the phylum and genus level of each group were reflected by color variation in clustering based on the similarity of abundance between samples. Because V3–V4 16S rRNA data have limited species-level resolution, putative species labels (e.g., *Eubacterium_xylanophilum*) are interpreted conservatively; functional inferences are anchored in well-described SCFA pathways rather than single-species claims.

### Gas Chromatography–Mass Spectrometry for the Determination of SCFAs

As described in the literature [35], pretreatment was performed by mixing 30 mg of colon contents with 300 μl of purified water. The treated samples were then subjected to GC/MS analysis using a 7890B gas chromatograph coupled with a 5977-mass selective detector (Agilent Technologies, USA). The temperatures of the injector, ion source, quadrupole, and GC/MS interface were set at 260, 230, 150, and 280oC, respectively, and the helium carrier gas flow rate was maintained at 1 ml/min. Mass spectrometry data was scanned at a frequency ranging from m/z 40 to 400 per second. Compound identification was confirmed through the infusion of pure standards and the comparison of retention times and corresponding mass spectra. The target ions (m/z) for acetic acid, propionic acid, butyric acid, isovaleric acid, and valeric acid were 117, 131, 145, 159, and 159, respectively. Data analysis was performed using the MassHunter program, and the content of SCFAs was externally standardized.

### Statistical Analysis

Analyses were performed based on the mean ± standard deviation of the data. One-way analysis of variance (ANOVA) and Tukey's multiple comparison test (GraphPad Prism software 7.0) were employed to calculate the significance between groups. *P* <0.05 were accepted as significant differences. Data from each experiment were duplicated at least three times.

## Results

### GR3 Alleviated the Symptoms of DSS-Induced Colitis

All mice in the blank group displayed a steady increase in body weight with regular feeding and drinking activities. As shown in Fig. 1B and 1C, the mice in the DSS group experienced decreased survival, weight loss, diarrhea and bleeding in the stool and anal after drinking 5 % DSS solution for 7 d, indicating the successful modeling of the mouse ulcerative colitis model. Intriguingly, in comparison with the DSS group, the weight of animals exhibited an increase in the GR3 medium and high dose groups as well as in the positive drug group (*P* < 0.01). Among them, no significant distinction was observed between the positive drug group and the GR3 high-dose group. For the evaluation of the severity of colitis, the DAI scores reflect the aggregate of weight change, stool tenderness and fecal bleeding in mice. A higher score indicates more severe colitis. As illustrated in Fig. 1D, a significantly higher DAI score was evidenced during the modeling period in the DSS group (*P* < 0.01), whereas it was substantially diminished after intervention with GR3 or positive drugs (*P* < 0.01).

The results illustrated that the colon of healthy mice had normally appeared and formed feces, whereas the colons were visible as dilute bloody feces in the DSS group, showing a significant shortening of the colonic length (Fig. 1E and 1F). Astonishingly, normal fecal morphology and reversal of the shortening of the colon associated with DSS were evidenced in the MES group as well as in the GR3 high and middle-dose administration groups. As shown in Fig. 1G and 1H, Histological scoring and H&E staining indicated well-arranged intestinal glands in the mucosal layer, without ulcerative lesions in the mucosa, intact epithelial cells lining the intestinal wall and without necrosis in the blank group. However, a majority of the mucosal fossa of the colonic mucosa with epithelium was destroyed in the DSS group, associated with inflammatory cell infiltration, whereby the inflammation invaded the submucosa. The lesions of the colonic mucosa were minimized in both GR3 and positive drug groups, resulting in diminished infiltration of inflammatory cells and enhanced epithelial and crypt integrity. In summarizing, GR3 markedly amended the symptoms of DSS-induced colitis in rodents, whilst maintaining the integrity of the colonic tissue against inflammatory cell infiltration. Therefore, GR3 dramatically attenuated DSS-induced mucosal lesions while mitigating pathological impairment in colitis mice.

### GR3 Regulated Inflammation Levels and the Intestinal Mucosal Barrier

**Fig. 2:**
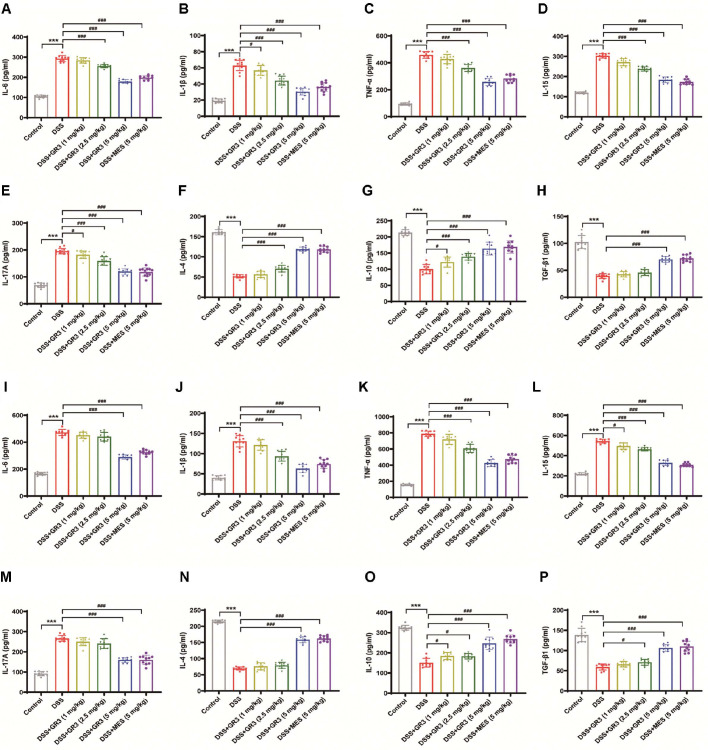
Ginsenoside Rb3 regulated suppressed inflammation infiltration in colitis mice. The levels of IL-6 (**A**) IL-6 (**B**) TNF-α (**C**) IL-15 (**D**) IL-17A (**E**) IL-4 (**F**) IL-10 (**G**) and TGF-β1 (**H**) in serum. The levels of IL-6 (**I**) IL-1β(**J**) TNF-α (**K**), IL-15 (**L**), IL-17A (**M**), IL-4 (**N**), IL-10 (**O**), and TGF-β1 (**P**) in colonic tissues of mice. All data are expressed as the mean ± SD (n = 10). *** *P* < 0.001. ^#^ *P* < 0.05, ^##^ *P* < 0.01, ^###^ *P* < 0.001.

Preliminary works have documented that significant upregulation of pro-inflammatory cytokines and downregulation of anti-inflammatory cytokines are synergistically associated with colitis[36]. As indicated in Fig. 2, significantly elevated levels of pro-inflammatory cytokines IL-6, IL-1β, TNF-α, IL-15, and IL-17A were obtained in serum and colonic tissues of mice in the DSS-group compared to the blank group (*P* < 0.001), whilst the levels of anti-inflammatory factors (including IL-4, IL-10, TGF-β1) were significantly decreased. Intriguingly, high doses of GR3 and MES dramatically lowered the levels of IL-6, IL-1β, TNF-α, IL-15, and IL-17A, whereas boosted the concentrations of anti-inflammatory factors (including IL-4, IL-10, TGF-β1) in serum and colonic tissues of mice.

**Fig. 3:**
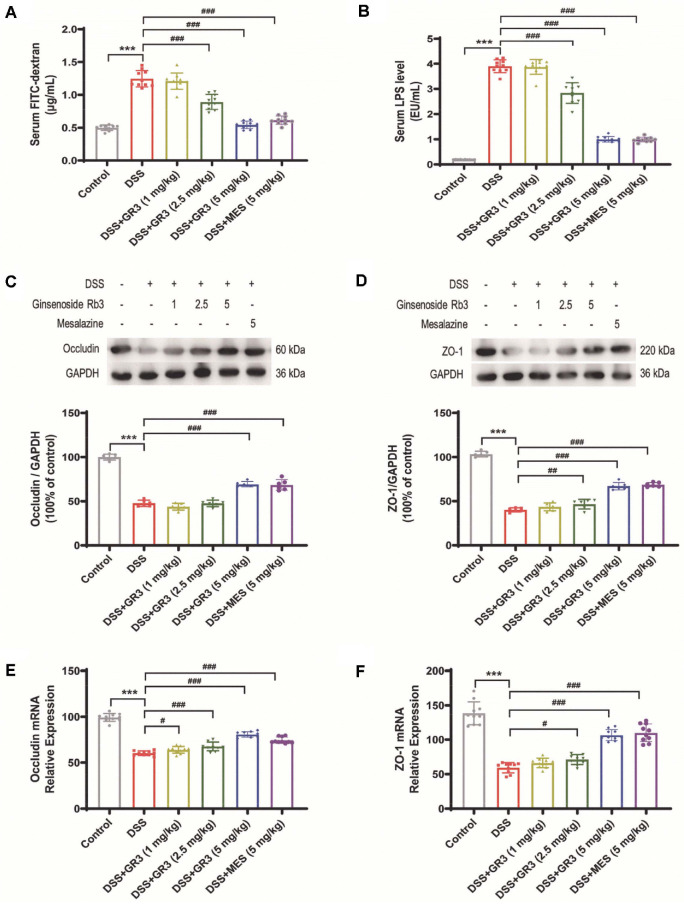
Ginsenoside Rb3 protected intestinal epithelial barrier dysfunction in colitis mice. (**A**) The levels of FITC-dextran in mouse serum. (**B**) The levels of LPS in mouse serum. (C-D) The protein levels of occludin and ZO-1 in colonic tissues of mice. (**E-F**) The mRNA levels of occludin and ZO-1 in colonic tissues of mice. All data are expressed as the mean ± SD (n = 10). *** *P* < 0.001. ^#^ *P* < 0.05, ^##^ *P* < 0.01, ^###^ *P* < 0.001.

Preliminary research has revealed that increased intestinal permeability in patients with ulcerative colitis is responsible for the entry of multiple curative factors into the bloodstream, contributing to the stimulation of the disease [37]. As shown in Fig. 3A, an increase of FITC-dextran levels in the serum of mice in the DSS group was observed, while GR3 administration significantly declined the level of FITC-dextran in the serum in a dose-dependent manner. Additionally, the occurrence of colitis in mice is characterized by an increment of LPS in the blood, which continuously stimulates the immune system to intensify the inflammatory response. To directly explore the influence of GR3 on DSS-induced colitis, serum LPS concentrations in mice were examined. As the results showed (as shown in Fig. 3B), mice in the DSS group displayed an increased level of LPS in serum, while GR3 administration efficiently diminished the level of LPS with dose dependence. In addition, as depicted in Fig. 3C-3F, compared with the blank group, a significant decrease of the tight junction protein of occludin and ZO-1 protein occurred in the DSS-group (*P* < 0.001), whilst both GR3 and MES administered ameliorated DSS-induced intestinal mucosal impairment in the DSS-group. According to findings, GR3 potentially modulated inflammation levels by enhancing intestinal tight junction protein expression to maintain the intestinal mucosal barrier in ulcerative colitis mice.

### GR3 Mitigated Liver and Kidney Impairment in Mice with Colitis

**Fig. 4:**
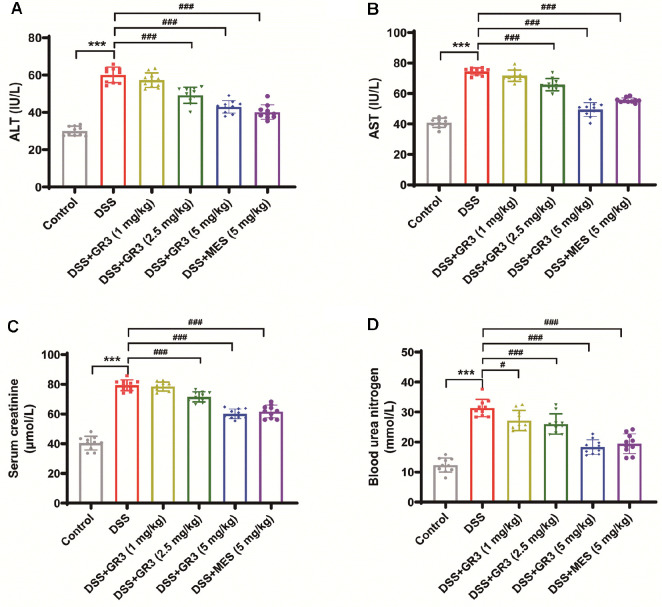
Ginsenoside Rb3 mitigated liver and kidney impairment in mice with colitis. (**A**) Changes of ALT. (**B**) Changes of AST. (**C**) Changes of serum creatinine. (D) Changes of blood urea nitrogen. All data are expressed as the mean ± SD (n = 10). *** *P* < 0.001. ^#^ *P* < 0.05, ^##^ *P* < 0.01, ^###^ *P* < 0.001.

Concerning the biochemical indicators correlated with hepatic and renal functions in mice, levels of serum AST and ALT reflected the severity of hepatocellular injury, while serum CR and BUN referred to the extent of renal cell injury. As revealed in Fig. 4A and 4B, levels of ALT and AST were statistically higher in the DSS group compared with the blank group (*P* < 0.01). Intriguingly, GR3 and positive drug intervention dramatically reversed the accumulation of AST and ALT in mice (*P* < 0.01). As indicated in Fig. 4C and 4D, compared with the blank group, the concentration of CR and BUN in the serum of the DSS group raised noticeably (*P* < 0.01). Intriguingly, with the intervention of GR3 and positive drug administration, both the levels of CR and BUN significantly declined (*P* < 0.01) with the therapeutic effect at a dose dependence of GR3. Collectively, these results illustrated that GR3 intervention notably inverted DSS-induced impairment of liver and kidney function in mice, safeguarding the role of GR3 in protecting liver and kidney function.

### GR3 Reshaped the Distribution of Intestinal Microflora in Rodents with Colitis

**Fig. 5:**
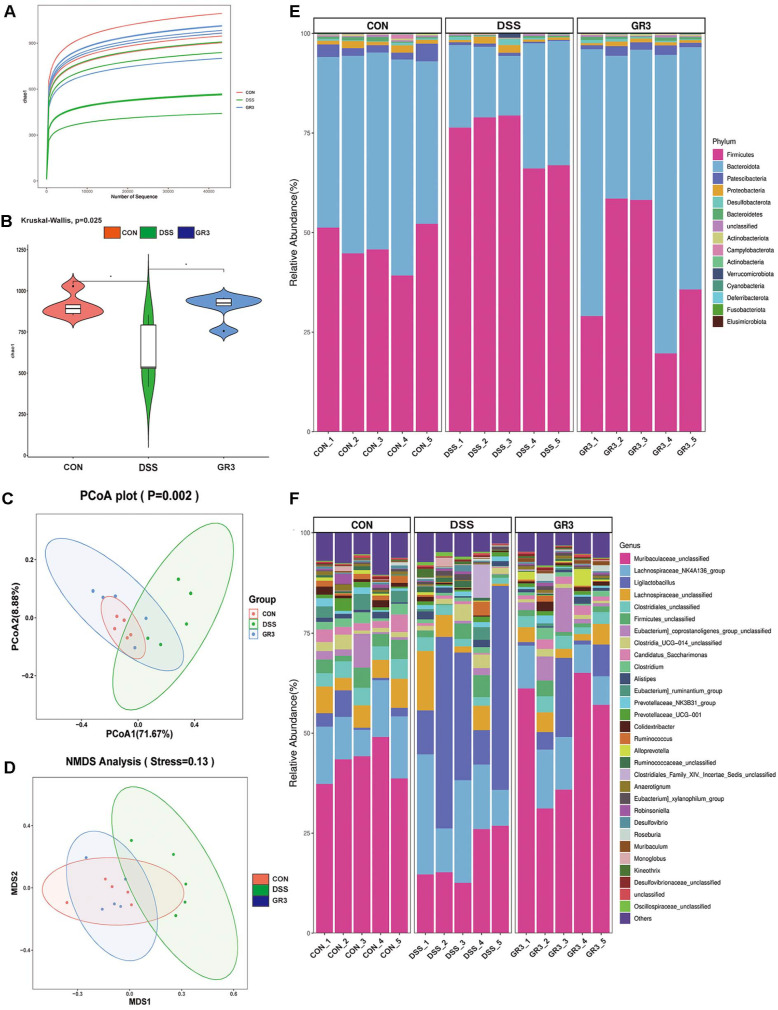
Ginsenoside Rb3 reshaped the distribution of intestinal microflora. (**A**) Rarefaction curves. (**B**) Chao1 index. (**C**) Principal coordinate analysis (PCoA). (**D**) NMDS analysis. (**E**) Difference of the relative abundance of phylum in mice. (**F**) Difference of the relative abundance of species in mice. All data are expressed as the mean ± SD (*n* = 5).

For measuring the composition of microbiota in mouse feces from the group of Control, DSS and GR3 (5 mg/kg), 16S rDNA sequencing to ascertain the impact of GR3 on DSS-induced murine colitis. For this study, the Rarefaction curve was employed to verify the species richness in samples with different amounts of sequencing data (Fig. 5A). Moreover, the abundance of intestinal bacteria species declined in the DSS group compared to the blank group, while the abundance of bacteria species was higher in the GR3 group (Fig. 5B). Meanwhile, Principal coordinate analysis (PCoA) and Non-metric multidimensional scaling (NMDS) are methods of descending multidimensional microbial data to depict the community differences between samples. In Fig. 5B and 5C, a more distant dispersion of the DSS group as compared to the blank group, signifying more changes of bacterial community structure in the DSS group than in the blank group, whilst GR3 intervention approaching the blank group, denoting that GR3 contributed to the restoration of intestinal flora abundance and structure.

**Fig. 6:**
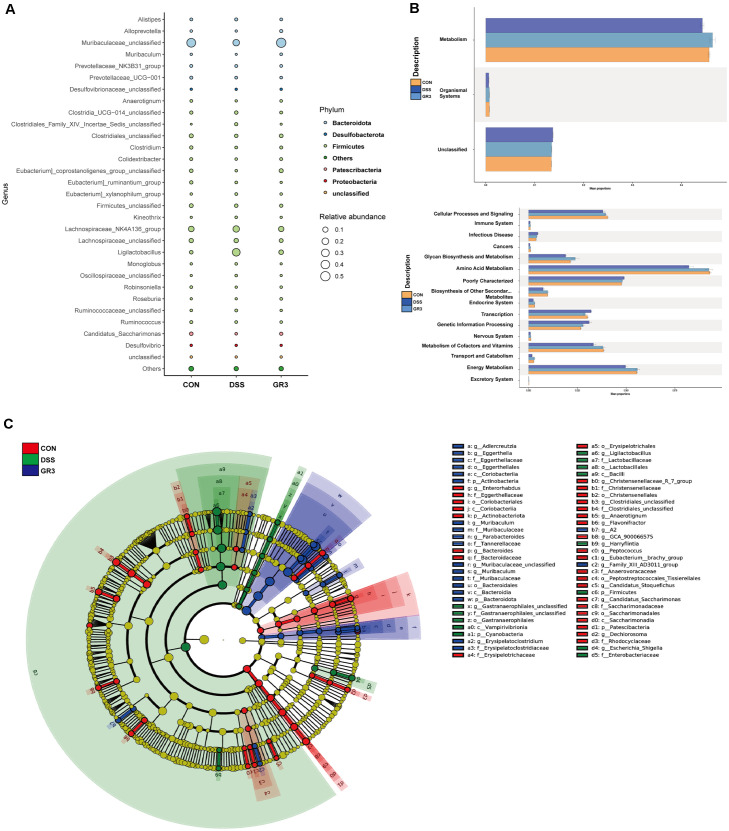
Functional prediction of ginsenoside Rb3 on intestinal microflora. (**A**) Bubble plot of the difference in microbial community composition. (**B**) PICRUSt2 pathways of function prediction were shown between groups. (**C**) LEfSe analysis of the difference in microbial community composition. All data are expressed as the mean ± SD (*n* = 5).

At the phylum level, as illustrated in Fig. 5E and S1A, the main 30 phyla at the phylum level indicated that *Bacillota*, *Bacteroidota*, *Patescibacteria* and *Proteobacteria* were relatively predominant. The relative abundance of *Bacillota* and *Cyanobacteria* was significantly higher in the DSS group compared to the blank group, while the relative abundance of *Bacteroidota*, *Patescibacteria*, *Desulfobacterota* and *Bacteroidota* was significantly lower. Compared with the DSS group, a significant decrease in the relative abundance of *Bacillota* and *Cyanobacteria* was observed in the GR3-treated group, while the abundance of *Bacteroidota*, *Patescibacteria*, *Desulfobacterota* and *Bacteroidota* was significantly elevated in the phylum. As shown in Figs. 5F and S1B, at the species level, an elevated abundance of *LachnosPiraceae_NK4A136*, *Ligilactobacillus*, *Clostridia_UCG-014*, *AlistiPes*, *Ruminococcaceae* and *Eubacterium_xylanoPhilum* were observed in ulcerative colitis mouse, which lowered the abundance of *Muribaculaceae*, *LachnosPiraceae*, *Clostridiales*, *Bacillota*, *Eubacterium_coProstanoligenes*, *Candidatus_Saccharimonas*, *Eubacterium_ruminantium*, *Prevotellaceae_NK3B31*, *Prevotellaceae_UCG-001*, *Clostridium_sP._Clone-17* and else. Intriguingly, GR3 critically reversed the variation of intestinal bacteria in mice with ulcerative colitis, encompassing an enhancement of *LachnosPiraceae_NK4A136*, *Ligilactobacillus*, *Clostridia_UCG-014*, *AlistiPes*, *Ruminococcaceae* and *Eubacterium_xylanoPhilum* abundance, while a diminution of *Muribaculaceae*, *LachnosPiraceae*, *Clostridiales*, *Bacillota*, *Eubacterium_coProstanoligenes*, *Candidatus_Saccharimonas*, *Eubacterium_ruminantium*, *Prevotellaceae_NK3B31*, *Prevotellaceae_UCG-001*, *Clostridium_sP._Clone-17* abundance. Unexpectedly, the relative abundance of *Ligilactobacillus* was significantly higher in the DSS-induced colitis group compared with controls. In addition, *Bacillus* was a minor taxon in absolute terms but showed a relatively higher proportion in the DSS group and declined after GR3 intervention, whereas *Lactobacillus* increased with GR3. We therefore report *Bacillus* as a low-abundance dysbiosis marker rather than a predominant commensal in this model. To pinpoint the distinct bacterial taxa from feces potentially serving as biomarkers at various levels, Linear discriminant analysis Effect Size (LEfSe), Bubble plot and PICRUSt2 KEGG pathway were performed to calculate microbial content between all groups and specific groups, which prompted the GR3 intervention significantly impinged on metabolic functions in the mice (Fig. 6A-6C). These results illustrated that the lowering level of pro-inflammatory factors in GR3 treatment was in association with the diminished abundance of *Bacillota* and *Cyanobacteria* in the phylum.

### GR3 Modulated the Content of Short-Chain Fatty Acids in the Murine Intestine

**Fig. 7:**
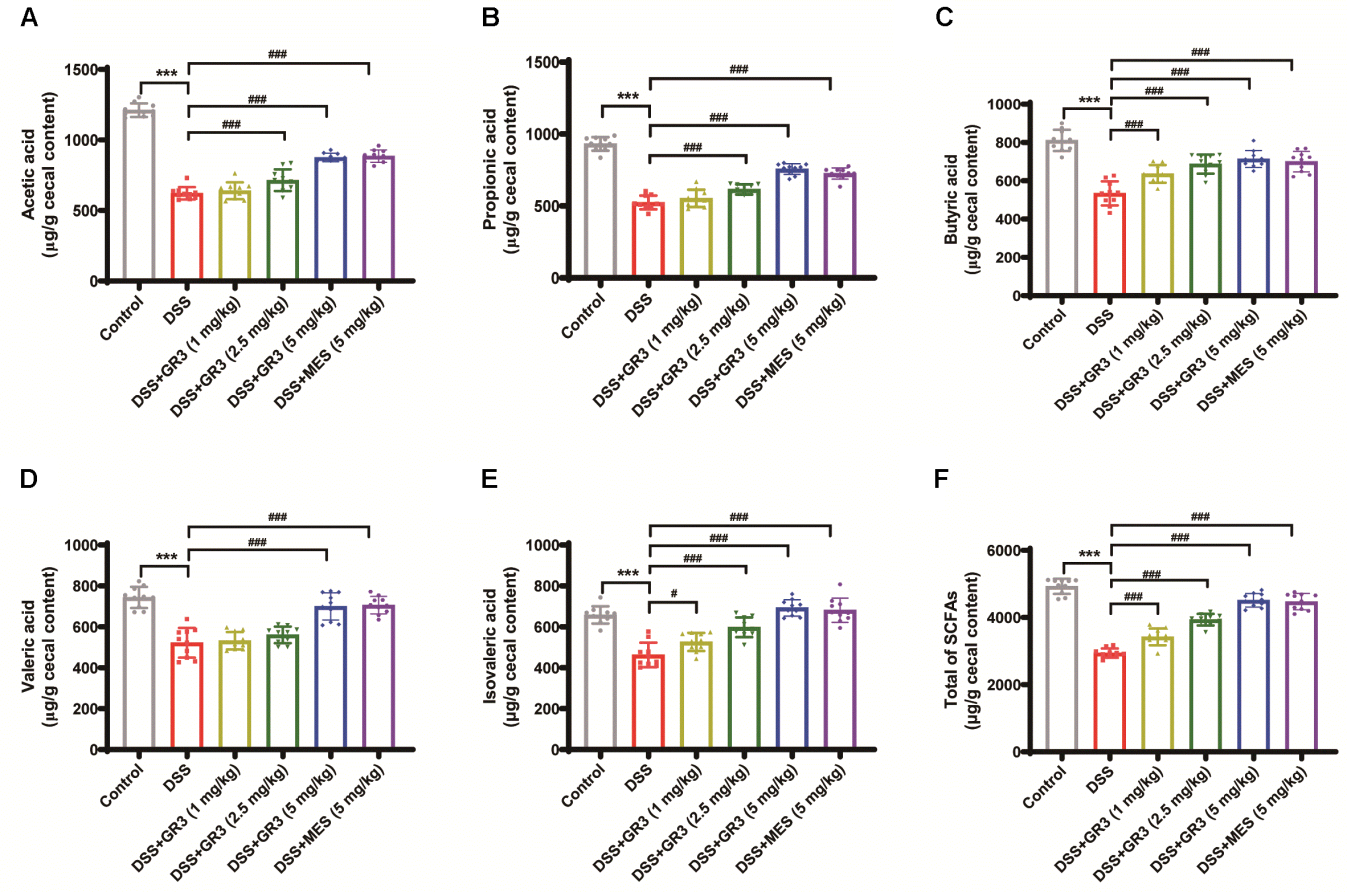
Ginsenoside Rb3 modulated the content of short-chain fatty acids in the murine intestine. Quantitation of the measurements of acetic acid (**A**) propionic acid (**B**) butyric acid (**C**) valeric acid (**D**) isovaleric acid (**E**) and total of SCFAs (**F**) levels in the feces sample of mice. All data are expressed as the mean ± SD (*n* = 10). *** *P* < 0.001. ^#^ *P* < 0.05, ^##^ *P* < 0.01, ^###^ *P* < 0.001.

As indicated in Fig. 7, The SCFAs mainly measured in mouse intestinal contents of each group were acetic acid, propionic acid, butyric acid, valeric acid and isovaleric acid, where acetic acid contained the highest amount and valeric acid the lowest amount. In comparison with that in the blank group, both the five SCFAs and the sum of SCFAs in the colonic contents of mice were significantly diminished in the DSS group. Intriguingly, in contrast to the DSS group, GR3 greatly elevated the concentration of the five short-chain fatty acids and total SCFAs in murine, whereas in a dose-dependent manner. Species-level alignment of SCFA producers. In our dataset, taxa compatible with acetate production were broadly present among *Bacteroidota*/*Bacillota*; propionate-linked lineages included *Bacteroides* (succinate/acrylate routes) and *Veillonellaceae*; and butyrate-linked lineages included *Lachnospiraceae*_NK4A136, *Ruminococcaceae*, and *Eubacterium_xylanophilum*. The concurrent rise of acetate, propionate, and butyrate with GR3 is therefore consistent with enhanced fermentative activity and cross-feeding, particularly acetate-dependent butyrogenesis. Therefore, it was concluded that GR3 counteracted a reduction in SCFAs in ulcerative colitis model mice.

## Discussion

**Fig. 8:**
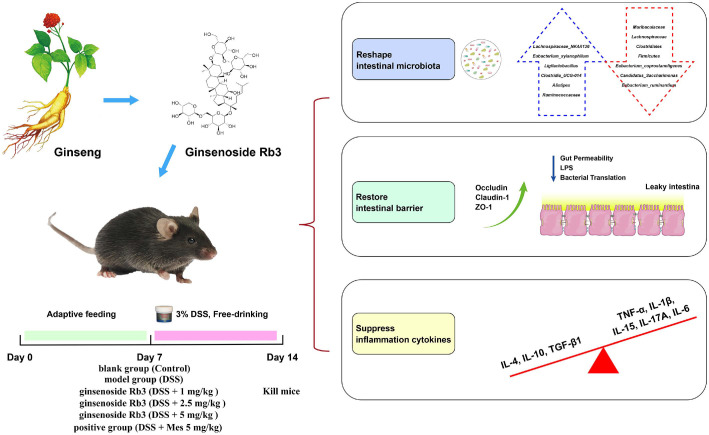
Schematic diagram of the protective effect of ginsenoside Rb3 in murine ulcerative colitis.

The present research has expanded our current understanding of UC and the intestinal microbiota, with a specific focus on mucosal dysfunction and microbiota dysbiosis. It aims to exploit the links and shared pathways between them, thereby offering an innovative approach for treating ulcerative colitis using the natural product GR3. In this study, we successfully used mice that were induced with 4% DSS to replicate major phenotypic changes observed in UC patients (Fig. 8). Our investigation sought to examine the anti-colitis effects of GR3 *in vivo*. This was evident through an increase in the DAI score, colon length, and histological scores, as well as a reduction in intestinal barrier dysfunction, inflammatory cell infiltration, and cytokine maturation. Furthermore, we explored the preventive effect of GR3 in a model of DSS-induced colitis, which resulted in increased microbial diversity and changes in composition. These changes countered the disruptions observed in the intestinal microbiota of the mice. In conclusion, these findings support the substantial potential of GR3 as a therapeutic agent for managing ulcerative colitis (Graphical Abstract). It achieves this by restoring the mucosal barrier and addressing the dysbiosis of the intestinal microflora.

As the initial epiphenomenon in the pathogenesis, an increasingly frequent explanation has attributed the emergence of the intestinal mucosal barrier to the progression of ulcerative colitis, while restoration of its integrity may mitigate the development of colitis [38]. As the largest organ of the human body in contact with the external environment, an integrated intestinal barrier is necessary in maintaining intestinal homeostasis and preventing and controlling diseases [39]. As the four parts of the intestinal tract, physical barrier, chemical barrier, immune barrier and biological barrier forms a comprehensive gastrointestinal barrier system to maintain homeostasis between internal and external environments of the organism [40]. Among them, the four primary barriers of the intestinal tract work as complementary wholes. Biologically, the intestinal barrier absorbs and digests water and nutrients from the body, while resisting attack by pathogens, foreign antigens and toxins [41]. In addition, the intestinal barrier is sensitive to multifactorial impacts, such as genetic susceptibility, diet, antibiotics, alcohol, and psychological stress, where intestinal barrier impairment has emerged as an indicator of UC deterioration [42]. Intriguingly, our study observed a significant increase in pro-inflammatory factors and a decrease in anti-inflammatory cytokines in the colon (*P* < 0.01). Compared to UC model mice, GR3 intervention dramatically reduced pro-inflammatory cytokine accumulation, demonstrating a positive correlation between GR3 modulation and cytokine release imbalance in UC. Furthermore, our investigation suggests that the therapeutic efficacy of Rb3 in UC may be closely related to its modulation of inflammatory cytokines, warranting further investigation into its underlying mechanisms of action.

While several ginsenosides, including Rg1, Rb1, Rh2, and Rk2, have shown therapeutic potential in UC models, GR3 offers unique mechanisms of action. Like other ginsenosides, GR3 exerts anti-inflammatory effects by reducing pro-inflammatory cytokines (TNF-α, IL-6) and promoting intestinal barrier integrity through upregulation of tight junction proteins. However, GR3 distinguishes itself by targeting the TLR4/MD2 complex, which inhibits NF-κB and MAPK pathways, a mechanism not prominently reported for other ginsenosides. Additionally, GR3 significantly reshapes the gut microbiota, enhancing the abundance of beneficial bacteria such as *Ligilactobacillus*, while other ginsenosides, such as Rg1, primarily modulate metabolic processes. Notably, GR3 demonstrated efficacy at lower doses (1, 2.5, and 5 mg/kg), suggesting that it may be more potent than other ginsenosides in UC treatment. These findings highlight the distinct and potentially superior therapeutic role of GR3, supporting its further investigation as a UC treatment.

Trillions of microorganisms in human intestines have been described as existing in symbiotic relationships with their hosts, fulfilling essential functions for healthy individuals such as nutrition, host defiance, and immune development [43]. Dysregulation of the enteric microbiome occurs naturally following disruption of its diversity, composition, or function, leading to negative effects on individuals, as evidenced by a loss of gut homeostasis and inappropriate immune activation. An imbalance of intestinal microflora leads to a thinning of the intestinal mucus layer, an inability to maintain the structure and function of the intercellular tight junctions, an expansion of the permeability of the epithelial cells, a diminution of the number of beneficial bacteria, and a proliferation of pathogenic bacteria, thereby triggering a cascade of inflammation as bacteria and toxins migrate through the barrier into the bloodstream, forming a vicious circle and exacerbating the damaging of UC [44, 45]. IBD patients are characterized by declining biodiversity (primarily of the *Bacillota*) decreased stability and elevated diversity of *Proteobacteria*, such as *Enterobacteriaceae* and *Bacteroides* [46]. Simultaneously, mucolytic and pathogenic bacteria tend to expand, contributing to a compromised mucosal barrier and enabling the invasion of more pathogens into the intestinal tissues. Various studies have documented the relevance of intestinal flora architecture to the pathogenesis of UC [47]. Additionally, the diversity and richness of the intestinal flora of UC patients was reduced, with the proportion of organisms including the *Bacillota* decreased and the proportion of bacteria such as *Aspergillus* and *Enterobacteriaceae* increased, depriving the intestine of the dynamic stability of the peripheral flora of UC patients [10]. Furthermore, with fecal microbiota transplantation from healthy individuals to patients with UC, a significant alleviation of UC symptoms was observed, pointing to an essential role of intestinal bacteria composition in the pathogenesis and treatment of UC [48]. In the present study, 16S rDNA sequencing was employed to characterize the composition of the intestinal flora, aiming to delineate the intestinal mechanism of GR3 in the treatment of UC. Similar to clinical IBD patients, dysbiosis ensued in the intestinal flora in the DSS-induced murine model of colitis, with decreased diversity and a higher relative abundance of *Bacillota* and Cyanobacteria, whereas the proportions of *Bacteroidota*, *Patescibacteria*, *Desulfobacterota*, and *Bacteroidota* declined in proportion. Dramatically, GR3 significantly elevated the abundance of *Bacteroidota*, *Patescibacteria*, *Desulfobacterota*, and *Bacteroides*, while significantly diminishing the accumulation of *Bacillota* and *Cyanobacteria* in the phylum. These results illustrated that the lowering level of pro-inflammatory factors in GR3 treatment was in association with the diminished abundance of *Bacillota* and *Cyanobacteria*. Although *Bacillus* is rarely predominant in the healthy gut, low-level increases can emerge under inflammatory and oxidative conditions due to barrier disruption and niche oxygenation; spore-forming/oxygen-resilient *Bacillales* may transiently gain a competitive edge. Given that GR3 tightened the epithelial barrier and dampened inflammatory tone, the ecological advantage for such taxa likely diminished, concordant with the reduced *Bacillus* ratio after GR3. In addition, V3–V4 16S has limited resolution for “*Bacillus* sensu lato”; thus, we avoid species-level claims and, where appropriate, reference its *Bacillaceae/Bacillales* context and emphasize robust phylum-level trends (*Bacillota*, *Bacteroidota*) together with functional SCFA increases.

Interestingly, in our study, the relative abundance of *Ligilactobacillus* increased in the DSS-treated group, which contrasts with the typical microbiota dysbiosis observed in UC, where beneficial bacteria are generally reduced. Recent studies suggest that, during early inflammation or epithelial barrier disruption, certain commensal lactic acid bacteria like *Ligilactobacillus* may temporarily proliferate in response to inflammation. This could be due to exposed epithelial adhesion sites and immune activation, favoring their growth. Furthermore, *Ligilactobacillus* has been linked to anti-inflammatory effects via SCFA production and GPR109A signaling. However, this increase may represent a transient shift rather than a beneficial change, and its long-term implications remain unclear. Further studies, such as SCFA quantification and microbial adhesion tests, are needed to determine whether this shift reflects a therapeutic restoration or a pathological response exacerbated by inflammation.

A major carbohydrate metabolite closely implicated in the development of ulcerative colitis following digestion by intestinal flora, SCFAs are responsible for stimulating ileal and colonic motility, enhancing intestinal mucosal blood flow and oxygen uptake, encouraging intestinal mucosal epithelial cell proliferation, avoiding atrophy of intestinal mucosal epithelial cells and glands, and initiating post-operative tissue repair, etc. [49]. Mainly consisting of acetic acid, propionic acid, and butyric acid, SCFAs represent an essential source of energy for the host, as an energy source for intestinal cells [50]. *Bacteroides*, *Bifidobacterium*, *Eubacterium*, *Streptococcus*, *Clostridium*, and *Streptococcus* are responsible for the manufacture of acetic acid. *Clostridium* is capable of generating propionic acid, while *Bacteroides*, *Eubacterium*, and *Clostridium* are capable of organizing butyric acid. Molecular biology revealed that acetic acid participates in lipogenesis and gluconeogenesis [51, 52], whereas acetic acid and butyric acid stimulate the short-chain fatty acid receptors GPR41 and GPR43, resulting in anti-inflammatory effects, while butyrate exerts beneficial properties on colonic epithelial cells and propionate on liver cells. Consequently, SCFAs, a fermentation component from intestinal microbiota, potentially facilitate the restoration of the gastrointestinal environment and modify the symptoms of ulcerative colitis in humans. The investigators have hypothesized the possibility of SCFAs originating from the intestine to enhance host health by decreasing the risk factors associated with inflammatory bowel disease and colorectal cancer. Accordingly, the present investigation investigated the alteration of SCFAs content in the colon of mice with ulcerative colitis following GR3 intervention treatment, intending to elucidate the therapeutic target of GR3.

Linking community shifts to SCFA profiles. Acetate is widespread among gut taxa and can prime butyrate formation via the butyryl-CoA:acetate CoA-transferase route, while propionate is classically generated by *Bacteroides* spp. and some *Veillonellaceae*. The broad SCFA increase we observed under GR3 aligns with (i) increased availability of fermentable substrates after barrier repair, and (ii) cross-feeding between primary degraders (acetate producers) and secondary fermenters (butyrate producers). Notably, the observed enrichment of *Lachnospiraceae_NK4A136*, *Ruminococcaceae*, and *Eubacterium_xylanophilum* is coherent with butyrate-producing guilds, whereas *Alistipes/Bacteroides* support propionate production and *Ligilactobacillus* can supply acetate/lactate as cross-feeding intermediates. We caution that V3–V4 16S constrains species-level certainty; hence, we interpret these assignments at the level supported by our data.

In our study the concurrent elevation of acetate, propionate and butyrate suggests a broadly activated fermentative microbiota rather than the selective recovery of a single SCFA-producing group. Microbial production of acetate is relatively widespread among gut taxa (*e.g.*, many *Bacteroidota* and *Bacillota* genera) and acts as a key precursor for cross-feeding. Propionate is classically generated by members of the *Bacteroidota* phylum (such as *Bacteroides* spp.) via the succinate or acrylate pathways, and by some *Veillonellaceae* from lactate/1,2-propanediol intermediates. Butyrate, in contrast, is produced by specialized *Bacillota* (*e.g.*, *Faecalibacterium prausnitzii*, *Roseburia* spp., *Eubacterium rectale*) via the butyryl-CoA: acetate CoA-transferase route and often depends on acetate as acceptor for cross-feeding. Consequently, the simultaneous rise in all three SCFAs in our DSS-colitis model may reflect (i) increased fermentable substrate availability (*e.g.*, liberated mucin/glycans due to barrier damage), (ii) enhanced cross-feeding between primary degraders (acetate producers) and secondary fermenters (butyrate-producers), and (iii) recovery or expansion of key SCFA-producing taxa after remission initiation. While the exact taxa responsible were not individually identified in this study, we have now included this mechanistic interpretation and emphasize the need for future work involving shotgun metagenomics, metabolite flux tracing and isotope labelling to map taxa-substrate-SCFA relationships. We also acknowledge this as a limitation and note that functionally linking taxon abundance to SCFA production *in vivo* remains a challenge in colitis models.

Our data indicate that GR3 tightens epithelial junctions (occludin/ZO-1), reduces LPS and pro-inflammatory cytokines, and raises acetate/propionate/butyrate, creating a niche less favorable to inflammation-adapted taxa and more permissive to barrier-associated commensals. Restoration of barrier function and inflammatory tone likely alters substrate gradients and cross-feeding (*e.g.*, acetate-dependent butyrogenesis), providing a host-mediated route for selective community shifts. In parallel, microbial deglycosylation of GR3 (a dammarane-type saponin) may yield metabolites with differential ecological effects, further biasing taxa composition. Compared with Rg1/Rb1/Rh2/Rk3, GR3 shares class-wide actions (anti-inflammation, barrier support, SCFA enhancement) yet in our model shows a distinct pattern—broad SCFA elevation and characteristic taxonomic reshaping (including *Ligilactobacillus*) at low daily doses (1–5 mg/kg)—underscoring GR3’s potential as a microbiota-modulating candidate for UC.

An innovative approach to clinical therapeutics for ulcerative colitis, which involves the use of natural phytochemicals to rebalance the intestinal microbiota for UC alleviation, is unveiled in this study. By harnessing the distinctive triterpenoid structure of GR3, this investigation offers a fresh perspective on the pharmacodynamics of traditional natural medicines, particularly focusing on traditional Chinese medicines, notably saponins. Intriguingly, the GR3 was first employed and exhibited an excellent therapeutic effect in the treatment of UC, which initially explored the molecular mechanism of GR3 in the treatment of UC and provided fundamental for subsequent development of GR3 as a promising new drug for UC. Admittedly, there are also some limitations in this study, including the impetration of validating the pharmacodynamic effects of GR3 in clinical patients with ulcerative colitis. In addition, the overall efficacy and safety of GR3 for the treatment of ulcerative colitis in humans has not yet been demonstrated, as well as the potential adverse side effects of GR3 in patients. Therefore, these findings underscore the need for continued emphasis on this aspect in future investigations.

## Conclusion

Collectively, the protective effects of GR3 on ulcerative colitis are strongly linked to the modulation of inflammatory cytokines and the composition of intestinal bacteria. This study elucidates the potential mechanisms by which GR3 ameliorates intestinal damage, regulates cytokine levels, and restores the balance of intestinal microflora. These findings provide a theoretical and experimental foundation for developing GR3 as a functional food or nutraceutical for the management of ulcerative colitis.

## Supplemental Materials

Supplementary data for this paper are available on-line only at http://jmb.or.kr.
