# Supplementary material for: Ginsenoside Rb3 Mitigates Murine Ulcerative Colitis by Modulating Intestinal Microflora and Short-Chain Fatty Acids
Source: J Microbiol Biotechnol. 2026 Jan 22;36:e2508035. doi: 10.4014/jmb.2508.08035 (PMC12883316; doi:10.4014/jmb.2508.08035)

J. Microbiol. Biotechnol. 2026. 36: e2508035

https://doi.org/10.4014/jmb.2508.08035

Supplementary Figure

Fig. S1. Ginsenoside Rb3 altered the relative abundance of gut microbiota at the phylum and genus levels. (A) Difference in the relative abundance of phylum between groups. **(B)** Difference in the relative abundance of genus between groups. All data are presented as the mean ± SD (*n* = 5).

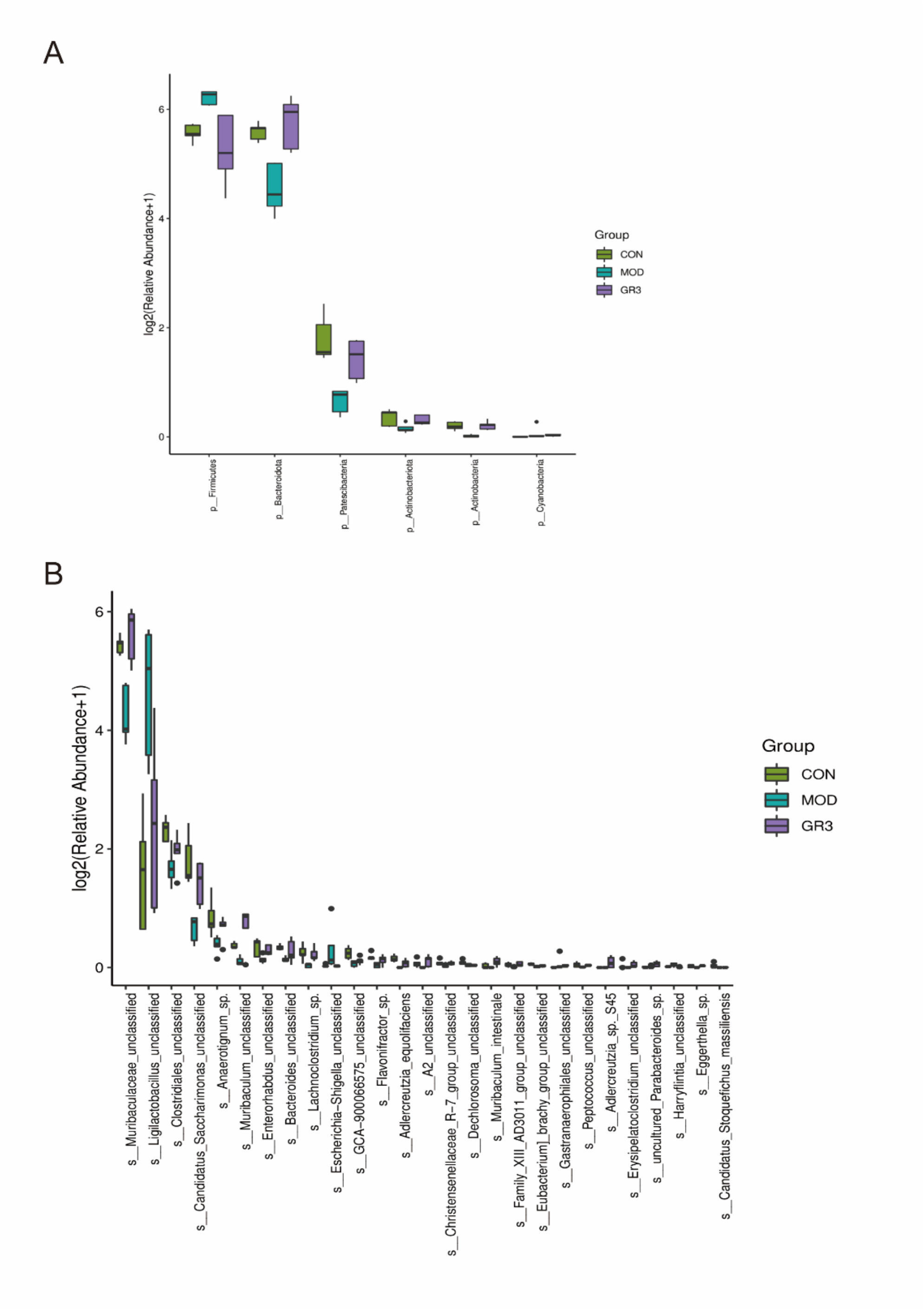

Supplement: Supplementary file 1 [file jmb-36-e2508035-supple.pdf]
